# Mini-Plication Surgery as a Valuable Tool for Surgical Treatment of Small Angle Residual Strabismus

**DOI:** 10.7759/cureus.98662

**Published:** 2025-12-07

**Authors:** Lakshmi K S, Deepti P, Krishnaprasad R

**Affiliations:** 1 Ophthalmology, Subbaiah Institute of Medical Sciences, Shivamogga, IND; 2 Ophthalmology, Mahipathi Madhwacharya Joshi Eye Institute, Hubballi, IND

**Keywords:** exotropia, mini-plication, prism diopters, residual strabismus, small angle strabismus

## Abstract

Introduction

Mini-plication is a relatively novel, minimally invasive surgical approach for the management of small-angle strabismus for small-angle residual strabismus. By creating a controlled fold of the extraocular muscle without detachment or excision, this technique preserves vascular supply and muscle integrity, allowing precise correction of minor deviations with reduced risk of complications compared to traditional procedures. Its technical simplicity and reversibility render it a promising option for patients requiring subtle ocular realignment. This study aims to evaluate the efficacy and safety of mini plication as a standalone intervention for small-angle residual strabismus.

Methods

A prospective analysis was performed involving nine patients (median age 66 years) who underwent mini-plication. The technique involved placing a 6-0 polyglactin suture through the central 3-4 mm portion of the muscle belly, situated nearly mm behind its insertion, and then advancing the suture through the sclera to secure a knot just in front of the muscle insertion. This creates a central fold that produces the needed plication effect.

Results

Patients presented with preoperative strabismus ranging from 10 to 16 prism diopters (mean, 12Δ). Postoperative deviations were reduced to a range of 2 to 8Δ (mean, 5.8Δ), yielding an average correction of 9Δ. No intraoperative or postoperative complications were observed.

Conclusion

Mini plication performed for small-angle residual strabismus under controlled anesthesia appears to be a safe, effective, and straightforward surgical alternative to prismatic correction for residual small-angle strabismus. This technique may be particularly beneficial for adult patients with residual deviations who seek a definitive treatment without reliance on spectacles.

## Introduction

Rectus muscle procedures, such as resection and full plication, which tighten the muscle, are used to treat strabismus with deviations of more than 15D. Both these strengthening procedures usually require either general anesthesia or local anesthetic block, such as peri-bulbar or retro-bulbar anesthesia. They comprise identifying and hooking the muscle, extensive posterior dissection of the muscle, and the resection or plication of the muscle, depending on the procedure chosen. Resection of muscle entails disinsertion, which disrupts the anterior ciliary blood supply and may cause anterior segment ischemia. One other major complication can be losing the muscle, which is catastrophic in inexperienced hands [1]. The full plication surgery for strabismus is advantageous when compared with the muscle resection surgery because total muscle disinsertion is not involved, and hence there is no risk of a lost muscle and nearly no risk of anterior segment ischemia. It is a reversible surgical procedure, especially when done within 5 days of surgery [2].

Evidence from iris fluorescein angiograms has proved beyond doubt that the full plication procedure is truly vessel sparing [2-3]. Plication procedure, however, corrects relatively larger deviations (>10 PD (prism diopters)) and needs peribulbar block anesthesia. Simple topical anesthesia will not suffice for an invasive full plication procedure [4]. Minimally invasive procedures like mini-tenotomy have been successfully performed under topical anesthesia [5]. But traditional strabismus surgeries, including recession and plications, require local or general anesthesia [6-8].

Here we have used a much less invasive technique that tightens the central part of a rectus muscle and corrects small-angle strabismus, which is usually residual strabismus.

## Materials and methods

This prospective, consecutive case study of mini-plication was approved by the MM Joshi Eye Institute, Hubballi institutional review board. Patients with incomitant strabismus were excluded from the study. Appropriate informed consent was obtained from adult patients and from the parents or legal guardians of pediatric patients prior to enrollment.

Patient (N=9) data, including demographics like age and sex, visual acuity, past ophthalmic history of previous ocular muscle surgery, symptomatic diplopia before and after surgery, and strabismic deviations before and after surgery, were collected.

Inclusion Criteria included consenting patients who were diagnosed with residual strabismus, with small angles of deviation (8 to 20 PD), and who were stable for at least 6 months. Exclusion Criteria included incomitant strabismus, previous adverse reaction or contraindication to anesthesia or surgery, presence of active ocular and/or systemic infection or inflammation.

The specific deviation angle, 8 PD to 20 PD, has been considered because this angle of deviation is considered too small for standard plication or recession procedures (>20 PD), but it is also too large for mini-tenotomy (< 4 PD). The surgical goal was to achieve a postoperative deviation of ≤5 PD, which would result in the resolution of diplopia, if present.

Strabismus measurements were obtained using the alternate prism cover test performed at a distance of 6 m and near at 33 cm. Surgical procedure, as explained below, was performed in the indicated consenting patients who fit our inclusion criteria. A single surgeon operated on all these patients to exclude bias.

Surgical procedure

The mini-plication was performed by isolating a 3 to 4 mm segment in the central portion of the rectus muscle. Either a limbal or forniceal approach was utilized to access the muscle. After conjunctival retraction, the central muscle segment was grasped with 0.5 mm toothed forceps, approximately 5 mm behind its insertion, taking care to avoid injury to the anterior ciliary vessels. After the forceps were carefully withdrawn from the sclera, a 6-0 double-armed polyglactin suture was threaded fully through the underlying muscle to anchor the chosen section. The suture was tied snugly around this area using a square knot, after which it was affixed to the sclera 0.5 mm anterior to the original insertion, thereby drawing the engaged muscle forward and creating central plication. The knot was secured, and the conjunctival incision was closed with absorbable sutures.

Patients were followed up monthly for a period of 6 months, and a careful history and angle of deviation were measured by the same ophthalmic consultant.

IBM SPSS software version 23 was used for data analysis. A binomial test was used to study the statistical significance of the results.

## Results

A total of nine patients within the age group, ranging from 8 to 62 years (median age: 44 years), underwent mini-plication surgery. Of the nine cases, two (22%) presented with hypertropia ranging from 4 PD to 10 PD, while seven (77%) had horizontal deviations, including six with esodeviation and one with sensory exotropia. An experienced strabismus surgeon did the mini-plication procedure.

Mini-plication resulted in an overall mean reduction in deviation of 8.22 PD (78.7%). Mean Preoperative Deviation: 10.44 ± 1.83 PD. Mean Postoperative Deviation: 2.22 ± 1.75 PD. When stratified by type of deviation, the following data were obtained as per Table 1.

**Table 1:** Mean reduction in prism diopters (PD) following mini-plication by the type of strabismus

Deviation	Mean Reduction (PD)	Standard Deviation(±)
Vertical deviation	5.75	2.9
Esodeviation	10.2	5.3
Exodeviation	8	0.7

The surgical goal was to achieve a post-surgical residual deviation within 5 PD in the 6-week follow-up period. This particular value was selected as 5 PD is within the fusional vergence range of both children and adults, thus can establish binocularity and alleviate symptoms in symptomatic diplopia patients. In our study, we found that we could achieve our surgical goal in 100% of patients. As shown in Table 2, preoperative deviation was measured in nine patients and compared to postoperative deviation using a paired-samples t-test. The mean preoperative deviation was 10.44 +/- 2.07 PD, which was significantly reduced to a mean postoperative deviation of 2.22 +/- 1.79PD. The mean correction achieved was 8.22 PD. This reduction in deviation was statistically significant (t=9.71, df=8, P < 0.0001), confirming the effectiveness of the mini-plication procedure in correcting strabismus.

**Table 2:** Pre- and postoperative deviation measures

	Pre Op Deviation (PD)	Post Op Deviation (PD)
Patient 1	10	0
Patient 2	12	4
Patient 3	10	4
Patient 4	8	0
Patient 5	10	0
Patient 6	14	2
Patient 7	8	4
Patient 8	12	2
Patient 9	10	4

## Discussion

Prismatic spectacles are commonly prescribed to alleviate diplopia in patients with small-angle strabismus; however, prisms do not address incomitant deviations and may be unsuitable for certain individuals, including those who have undergone refractive surgery and prefer to avoid dependence on glasses. The mini-plication technique described here targets deviations ranging from 8 to 10 PD, which fall below the typical threshold for conventional surgical correction. Although further investigation is necessary to accurately define the dose-response relationship, our observations indicate a correction of 5 to 7 PD in patients without prior surgery, and 8 to 10 PD per muscle in those who had antagonist muscle recession. The magnitude of correction may be modulated by adjusting the position of the securing suture relative to the muscle insertion. This procedure appears particularly effective for small esodeviations, which not only cause pronounced diplopia but can also lead to amblyopia in pediatric patients. These results align well with previous reports describing mini-plication as a minimally invasive, precise, and well-tolerated procedure that can be performed under topical anesthesia, making it particularly suitable for adult patients experiencing diplopia [9].

Compared to traditional strabismus operations, which necessitate the isolation and removal of the muscle, the mini-plication technique offers numerous benefits. Mini-plication differs from traditional muscle resection in that it involves folding and suturing the muscle belly without disrupting the entire muscle insertion. This approach preserves anterior ciliary circulation, reducing the risk of anterior segment ischemia, a rare but serious complication reported with larger resections [10-11]. Additionally, it avoids the risk of lost muscle and allows for potentially earlier reversibility, with decreased postoperative inflammation and bleeding [12]. These advantages support mini-plication as an important addition to the strabismus surgeon's toolkit, especially in cases where small but significant alignment improvements are required.

Compared to other minimally invasive options like mini-tenotomy and standard plication, mini-plication fills a treatment niche for deviations between 8 and 20 PD, too large for mini-tenotomy alone but smaller than conventional recession or resection candidates [4]. The correction magnitude observed in this study is consistent with the range reported in the literature, further validating mini-plication’s effective dose-response relationship [13-14].

The absence of significant complications or overcorrections in this study supports the procedure’s safety profile, consistent with other reports indicating reduced tissue trauma and scar formation [15-16].

This study has several limitations that should be considered when interpreting its findings. First, the small sample size (n=9) limits the statistical power and the ability to detect rare adverse events or subtle differences in outcomes. Second, the short-term follow-up does not provide information about the long-term stability of ocular alignment, potential late-onset complications, or recurrence rates. Third, as a single-center, non-randomized case series without a control or comparison group, the results are subject to selection bias and may not be generalizable beyond this specific population and surgical setting. Additionally, the study population consisted exclusively of adults with residual small-angle exotropia, limiting the applicability of these results to pediatric or primary large-angle cases. Finally, while the study reports on deviation correction, there is an absence of standardized outcome measures. Specifically, the lack of a widely validated, uniform tool for measuring diplopia (double vision) and patient-reported outcomes (PROs) restricts the comparability of our results with other published literature and may introduce variability in the subjective findings reported. These factors underscore the need for larger, multicenter, randomized studies with long-term follow-up to more definitively determine the efficacy and safety of mini-plication for small-angle strabismus.

## Conclusions

Mini-plication offers a targeted, minimally invasive surgical approach for patients with small-angle strabismus, effectively reducing ocular deviation while minimizing tissue disruption. This technique meets surgical goals with a relatively high success rate and relatively low complication profile, enhancing patient outcomes such as diplopia resolution and ocular alignment. Its role as an alternative to more extensive muscle surgeries is particularly valuable in cases where the angle of deviation is moderate and precise correction is needed. Continued studies with larger cohorts and longer follow-up will help solidify its place in strabismus management.
